# Advances in 3D Gel Printing for Enzyme Immobilization

**DOI:** 10.3390/gels8080460

**Published:** 2022-07-22

**Authors:** Jialong Shen, Sen Zhang, Xiaomeng Fang, Sonja Salmon

**Affiliations:** Department of Textile Engineering, Chemistry and Science, Wilson College of Textiles, North Carolina State University, Raleigh, NC 27695-8301, USA; jshen3@ncsu.edu (J.S.); szhang53@ncsu.edu (S.Z.)

**Keywords:** 3D printing, biocatalysis, biosensing, enzyme, hydrogel, immobilization

## Abstract

Incorporating enzymes with three-dimensional (3D) printing is an exciting new field of convergence research that holds infinite potential for creating highly customizable components with diverse and efficient biocatalytic properties. Enzymes, nature’s nanoscale protein-based catalysts, perform crucial functions in biological systems and play increasingly important roles in modern chemical processing methods, cascade reactions, and sensor technologies. Immobilizing enzymes on solid carriers facilitates their recovery and reuse, improves stability and longevity, broadens applicability, and reduces overall processing and chemical conversion costs. Three-dimensional printing offers extraordinary flexibility for creating high-resolution complex structures that enable completely new reactor designs with versatile sub-micron functional features in macroscale objects. Immobilizing enzymes on or in 3D printed structures makes it possible to precisely control their spatial location for the optimal catalytic reaction. Combining the rapid advances in these two technologies is leading to completely new levels of control and precision in fabricating immobilized enzyme catalysts. The goal of this review is to promote further research by providing a critical discussion of 3D printed enzyme immobilization methods encompassing both post-printing immobilization and immobilization by physical entrapment during 3D printing. Especially, 3D printed gel matrix techniques offer mild single-step entrapment mechanisms that produce ideal environments for enzymes with high retention of catalytic function and unparalleled fabrication control. Examples from the literature, comparisons of the benefits and challenges of different combinations of the two technologies, novel approaches employed to enhance printed hydrogel physical properties, and an outlook on future directions are included to provide inspiration and insights for pursuing work in this promising field.

## 1. Introduction

High reaction selectivity at mild operational conditions makes enzymes and biological catalysts desirable for use in modern industrial chemical processes [1]. Attaching or entrapping enzymes on or in solid supports (called “immobilization”) facilitates their recovery and reuse, improves stability, enhances longevity, broadens applicability, and reduces overall processing and chemical conversion costs [2,3,4,5]. Many different support materials (or “carriers”) have been developed for enzyme immobilization to match specific process requirements and expand industrial-scale deployment of biocatalysts for applications that would otherwise not be feasible with dissolved (or “free”) enzymes [6,7,8,9,10,11,12]. Fundamentally, immobilization increases the size of the biocatalyst, making it easier to hold and recover. Small carrier particles are commonly used for immobilization because these can be dispersed in the liquid reaction medium to enhance mixing, which helps speed up reaction kinetics. However, this approach subsequently requires separation processes to recover the biocatalysts, such as filtration, centrifugation, or magnetic attraction [13]. Conventional immobilization carriers made as beads and granules for packed-bed reactors do not lend themselves to forming complex 3D structures with the unique shapes, sizes, and physical properties needed to produce customized biocatalytic reactor components. An emerging solution to this challenge is three-dimensional (3D) printing, also known as additive manufacturing. Three-dimensional printing is capable of rapidly producing complex shape designs with precise dimensions using diverse materials and has been commercialized for many applications. Depending on the 3D printing technique and materials used, resulting structures can be rigid or flexible, hydrophobic or hydrophilic, or any combination of these, along with other desirable properties, such as water-permeable gel environments. Immobilizing enzymes on or within 3D printed materials enables high-resolution placement of enzymes in complex structures, simple scale-up with minimal material waste, and continuous-flow well-mixed reaction geometries without laborious separation steps while retaining all the benefits provided by conventional immobilized enzymes [14,15].

The basic design principle of 3D printing is to digitally slice a 3D object into multiple 2D thin layers and then recreate that object by depositing materials in the 2D pattern one layer at a time. This design approach is well-suited for enzyme immobilization. Currently, there are seven main 3D printing technologies: material extrusion (ME), vat photopolymerization (VP), powder bed fusion (PBF), material jetting (MJ), binder jetting (BJ), sheet lamination (SL), and directed energy deposition (DED) [16,17,18,19]. Among these technologies, ME and VP are the two most common methods used for enzyme immobilization. Enzymes can either be immobilized onto a completed structure (post-printing) or can be incorporated into the printing materials during structure fabrication (entrapment). In post-printing, enzymes are covalently attached or physically coated onto pre-made 3D printed supports, which can be made from a wide variety of printing materials. Since the enzyme immobilization process is carried out separately from 3D printing, no adaptation of the 3D printing process is required. In contrast, the entrapment method requires careful consideration of enzyme compatibility with the raw printing materials, which directly influences printability [20,21,22,23,24] and enzyme activity. While post-printing immobilization is applicable for all 3D printing technologies, this method usually requires harsh chemicals for surface functionalization and time-consuming multi-step immobilization processes. Alternatively, enzyme entrapment can be carried out by simple one-step fabrication methods that result in high enzyme activity retention as a result of the mild immobilization conditions. A drawback is that the choices of materials that are compatible with enzymes for one-step entrapment are limited but growing.

Three-dimensional printing recipes that incorporate hydrogel components—polymers capable of absorbing large amounts of water while maintaining a solid form—provide especially compatible environments for direct enzyme inclusion by entrapment. Traditional hydrogels suffer from poor mechanical properties and severe mass transfer barriers that slow the transport of reaction substrates and products through the gel. Now, new bioink materials, mechanical reinforcement strategies, and finer structures are being developed to overcome these common drawbacks, presenting a promising paradigm change for the future of enzyme immobilization. Major advantages, challenges and future developments in using free enzymes, conventional immobilized enzymes and 3D printed immobilized enzymes are compared in Table 1. Enzyme immobilization by 3D printing is actually a subcategory within the broader field of enzyme immobilization, meaning that conventional immobilization and 3D printing immobilization are not mutually exclusive—they share many common features. What 3D printing especially offers is easy design and fabrication of complex structures. These can be tailored for enzyme immobilization purposes that cannot be achieved in other ways. Therefore, any improvements made upstream (left side of Table 1) can also benefit the development of 3D printed biocatalyst products. For example, improving enzyme stability by protein engineering will benefit all enzyme immobilization techniques and new innovations in conventional immobilization chemistries will also help improve 3D printed immobilization.

The objective of this review is to promote the convergence of research between conventional enzyme immobilization and 3D printing techniques, with special emphasis on the importance and versatility of gelation mechanisms for achieving sophisticated functionality under mild fabrication conditions. Gel matrices provide favorable hydrated microenvironments for optimal enzyme activity and have unique physical properties that are essential for certain applications, such as tissue engineering and permeable sensors. Practical insights on materials and methods are provided as a selection guide for both new and seasoned researchers. The analysis focuses on enzyme and material interactions rather than on the broader role of 3D printing in making tools, scaffolds, implants, enclosures, and other devices. After briefly introducing conventional enzyme immobilization and the working mechanisms of the two most commonly used 3D printing methods, the discussion of the current literature is divided into two main immobilization categories. The post-printing category is organized according to the major 3D printing technologies used, because once the material is printed, enzyme immobilization is carried out by common techniques. The entrapment category is organized according to the physical state of the entrapping matrix, with gel printing further divided based on gelation mechanisms. The creation of gel structures with high-resolution and precise combinations of size, shape and fabrication materials, controlled throughout the structure from sub-micron to macro-scale, is an exciting design innovation space for which mild 3D printing mechanisms are uniquely equipped and superior to conventional immobilization approaches. Following an acknowledgement of the limitations of this review, the conclusions and future perspectives present development trends observed in the most recent publications, along with predictions on areas of significant future growth. 

### 1.1. Conventional Enzyme Immobilization Methods

Cell-free biocatalysis provides numerous benefits over whole-cell systems and enzyme immobilization technology represents a key solution for its large-scale industrial implementations [25]. Most enzyme immobilization methods involve the use of solid carriers or support materials. Exceptions are the well-known carrier-free crosslinked enzyme aggregates (CLEA) and crosslinked enzyme crystals (CLEC), where the particles are composed entirely of chemically crosslinked enzymes [26]. Support materials come in different sizes and shapes, such as thin films, microbeads, nanoparticles, and nanofibers [27], and can be prepared before enzyme immobilization or during enzyme immobilization. Immobilization during the formation of materials includes physical entrapment or encapsulation. The enzyme is retained because it is physically larger than the pore size of the entrapping materials. Immobilization after material formation mainly consists of physical adsorption, covalent attachment, and affinity binding [28]. In this case, physical or chemical binding interactions between the enzyme and the support surface are essential. Such binding can be very effective and selective when the enzyme contains specific groups that interact favorably or bond irreversibly with the support material surfaces. For example, through protein-fusion or site-directed mutagenesis, specific material binding domains, tags or anti-tags, and modifiable amino acid residues can be incorporated into the enzyme structure for controlled enzyme immobilization [29,30,31]. Another more common strategy is to chemically functionalize or activate the support material surface to react with amino acid residues naturally occurring in the enzyme. The most commonly targeted residue is lysine. Lysine’s pendant amino group forms imine linkages (Schiff’s base) with aldehydes and forms amide bonds with carboxyl groups, facilitated by carbodiimide activated esters [32]. As discussed further in Section 2, glutaradehyde (GA) and 1-ethyl-3-(3-dimethylaminopropyl)carbodiimide with N-hydroxysuccinimide (EDC/NHS) are frequently used to functionalize 3D printed support materials to be amine reactive. On-going innovations in the chemistry of conventional enzyme immobilization can be directly adopted in the post-printing enzyme immobilization scenario.

### 1.2. 3D Printing Basics

The two 3D printing techniques most relevant for enzyme immobilization are material extrusion and vat photopolymerization. Fundamentally, ME requires high viscosity raw material liquids while VP requires low viscosity liquids.

#### 1.2.1. Material Extrusion

**Figure 1:**
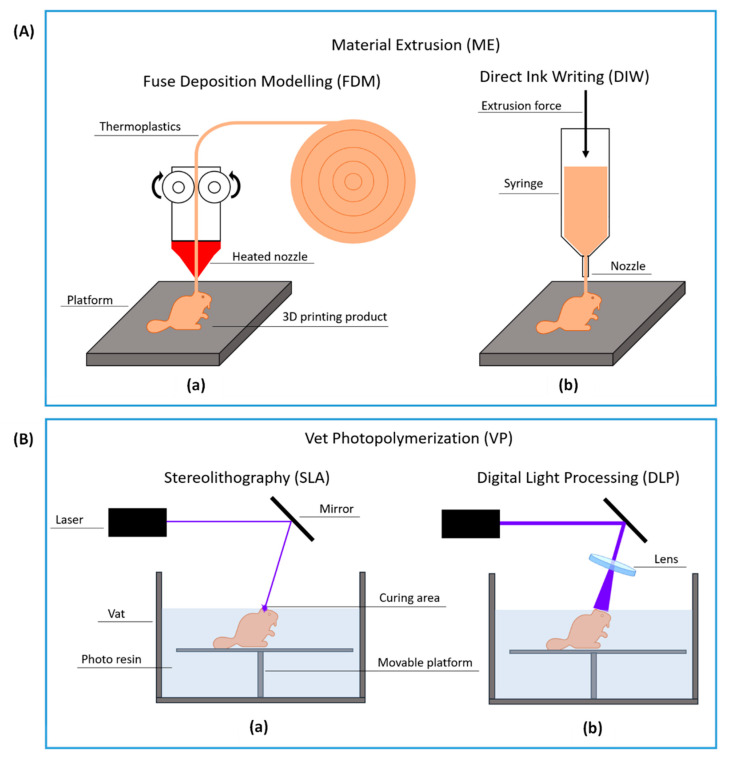
Common 3D print methods. (**A**) Material extrusion (ME): (**a**) fused deposition modeling (FDM) and (**b**) direct ink writing (DIW) (**B**) vat photopolymerization (VP): (**a**) stereolithography (SLA) and (**b**) digital light processing (DLP).

ME works by extruding fluid raw material through an extrusion head nozzle, past which the material solidifies onto the platform or onto the surface of the preceding layer by various mechanisms, including changes in temperature, shear rate, or on-set of crosslinking reactions. Meanwhile, the extrusion head or the platform moves, following a predetermined path, to deposit one layer of materials. The process then continues to stack materials layer-by-layer until the whole object is formed. Based on raw material physical properties, two different mechanisms for ME are followed: (1) fused deposition modeling (FDM) and (2) direct ink writing (DIW) [33,34,35,36]. FDM involves the melting and deposition of thermoplastics and therefore requires heating the print head. As shown in Figure 1Aa, the thermoplastic filament is dragged by one pair of rollers and melted by the heated nozzle. The polymer melt solidifies swiftly once it is deposited on the platform at ambient temperature. Raw materials used for FDM are in the form of thermoplastic filaments, polymer powders or pellets. Because the high extrusion temperature denatures (inactivates) most enzymes, post-printing immobilization is the preferred method for immobilizing enzymes on supports formed by FDM [37,38,39,40,41,42]. As shown in Figure 1Ab, DIW uses pneumatic or mechanical force to extrude a wide variety of fluidic materials. Relevant fluids include polymer solutions, shear-thinning gels and colloidal materials. DIW usually does not require a heated nozzle and is therefore more amenable for immobilizing enzymes by entrapment [43,44,45].

#### 1.2.2. Vat Photopolymerization

VP uses a vat of photosensitive liquid material and a light source to solidify liquid materials layer-by-layer to form a 3D structure. As shown in Figure 1Ba, ultraviolet (UV) light generated by a laser source is reflected by a movable mirror that is used to control the light path directed to the liquid materials. The top layer of photo-curable resin receives energy from the UV light and polymerizes to form a solid layer. After one layer completes curing, the platform moves down to let uncured resin flow over the cured part. A new layer is then cured with the same process. Two common VP methods are used to immobilize enzymes: (1) stereolithography (SLA) and (2) digital light processing (DLP). SLA generates a point or line light guided by a movable mirror to cure the photo resin top layer where the light lands. In contrast, DLP uses a digital light processor that can directly generate the whole pattern for one layer at a time. (Figure 1Bb) The small 2D light pattern is enlarged by a lens, and the actual 2D pattern is projected onto the surface of the resin causing the whole layer to cure simultaneously. Because VP does not require a high temperature to melt materials or high material viscosity to hold a shape for curing, enzymes can be directly mixed with photo-curable resin and immobilized by an entrapment [46]. Nevertheless, post-printing enzyme immobilization is also a common practice with VP methods, leveraging its high spatial resolution to create fine structures [47,48]. The 3D print scaffold can be generated first and then the enzyme is immobilized by a covalent reaction or physical coating. 

## 2. Post-Printing Immobilization

The strategy of immobilizing enzymes after fabricating the support materials essentially divides the whole process into two non-interfering steps and is technically applicable to all types of 3D printing techniques. In other words, the selection and optimization of the 3D printing process can be made largely without considering enzyme stability condition limits. Subsequently, the selection of enzyme immobilization method is mainly concerned with finding a suitable surface modification method applicable to the surface chemistry of the printed support materials, for which a wealth of literature exists [28,32,49]. Recent studies utilizing post-printing immobilization are summarized in Table 2.

### 2.1. Fused Deposition Modeling

**Figure 2:**
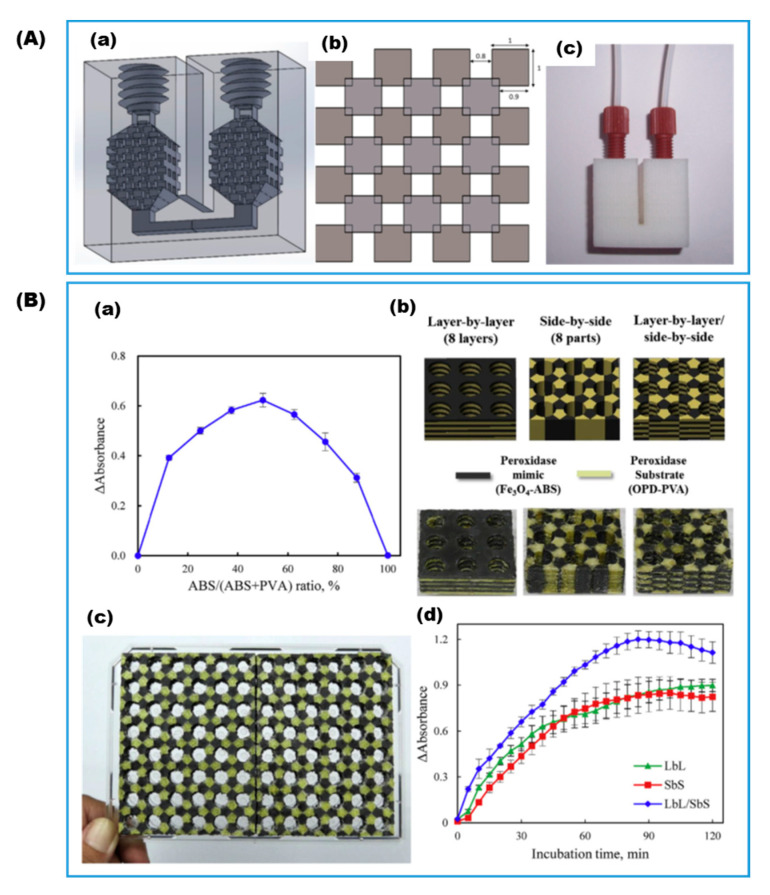
(**A**) ABS-based flow reactor: (**a**) CAD drawings, (**b**) ordered cuboids pattern in the reaction chamber, and (**c**) photo of the printed reactor (Reproduced with permission from Ref. [20]); (**B**) multi-material substrate-impregnated micro-titer plate for glucose determination: (**a**) effect of ratio of GOx, peroxides mimic, and substrate on signals as varied by different 3D patterns, (**b**) CAD drawings and photos of plates with different material stacking pattern, (**c**) photo of two printed 48-well plates fitting to a regular 96-well plate frame, and (**d**) effect of 3D pattern on the kinetic signals (Reproduced with permission from Ref. [22]).

Due to its accessibility, material extrusion by FDM is among the most popular general-use 3D printing techniques for rapid prototyping in the research and development of biocatalytic reactors, biosensors, and bioanalytical tools. With few exceptions, most published studies using FDM support materials for enzyme immobilization applied enzymes to the objects after they were printed. As one of the early adopters, Su et al. [20] fabricated a flow bioreactor (Figure 2A) using ABS filament and immobilized glucose oxidase and lactate oxidase on its surface through glutaraldehyde activation and covalent attachment. The device carried out oxidation of sampled rat brain microdialysate for in vivo dynamic monitoring of glucose and lactate. In a later development, Su et al. employed a dual print head FDM incorporating iron oxide nanoparticles. These particles exhibited peroxidase-like catalytic activity and served as an enzyme-mimic or surrogate in the ABS matrix and as a chromogenic substrate in a second polyvinyl alcohol (PVA) matrix [22]. The printed multi-material object took the shape of a standard 96-well microtiter plate (Figure 2B), with glucose oxidase later immobilized by drop-casting in each well for rapid colorimetric determination of glucose.

Post-printing enzyme immobilization coupled with FDM printing is broadly applicable to various filament materials regardless of their melting temperatures. For example, Peris et al. [37] printed a continuous-flow microfluidic bioreactor using nylon filament and treated its surface consecutively with 5M HCl and glutaraldehyde to achieve surface covalent immobilization of ω-transaminase. Immobilized enzyme showed comparable activity to the free enzyme and was able to maintain activity for a total of 105 catalytic cycles during which optically pure chiral amines were produced from ketones. 

**Figure 3:**
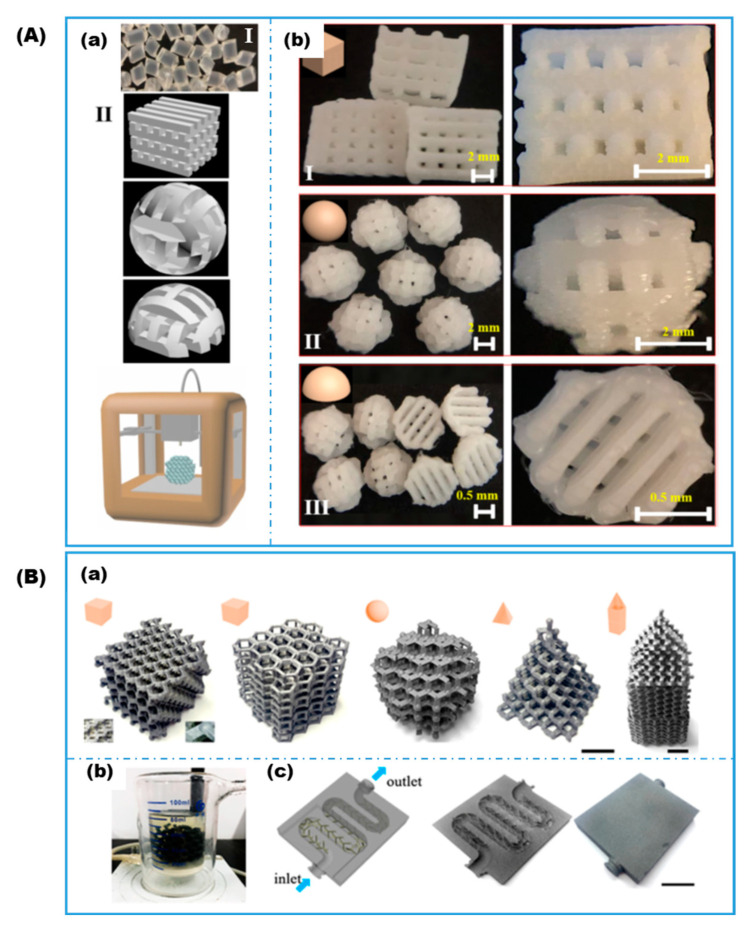
(**A**) The 3D-printed PLA-based macroscaffolds for enzyme immobilization: (**a**) as-received PLA (I) and CAD drawings of the designed scaffolds (II) and (**b**) photos of the 3D-printed scaffolds (reproduced with permission from Ref. [42]). (**B**) The 3D-printed carbon fiber reinforced PLA-based scaffolds for enzyme immobilization: (**a**) CAD images of 3D scaffolds; (**b**) photo of an integrated reactor with immobilized enzymes on 3D-printed scaffold; and, (**c**) 3D models of microfluidic reactors with scaffolds in the channel (reproduced with permission from Ref. [56]).

Another popular material used in FDM is poly(lactic acid) (PLA) or PLA-based composite filament. Zhang et al. [42] fabricated several PLA-based macro-scaffolds (Figure 3A) for the physical adsorption immobilization of lipase, which exhibits superactivity when immobilized on hydrophobic carriers. Activity recovery of up to 137.3% was achieved with phenyl group functionalized surfaces in comparison to only 6.0% for the native PLA surface. The immobilized lipase retained stable activity over nine repetitive usage cycles and produced high purity (S)-1-indanol, which is an essential intermediate compound for pharmaceutical synthesis. Ye et al. [56] fabricated and chemically modified scaffolds of different shapes, including cube, sphere, and pyramid, and microfluidic reactors using carbon fiber reinforced PLA filament as a versatile framework for immobilizing various enzymes through both covalent attachment and physical adsorption (Figure 3B). The immobilized enzymes retained 88–92.8% of their original activities after 10 reaction cycles. Because of its electrical properties, carbon-based PLA filament has been widely used to make electrodes for sensors and fuel cells. Pumera [41,50,51,52] and co-workers 3D printed a series of electrodes using graphene/PLA or carbon black/PLA composite filament for immobilization of various enzymes, which confer bio-recognition properties to the biosensors. To improve the electroconductivity, the insulating PLA phase was digested enzymatically with proteinase-K, or treated with a combination of solvent, sonication, and electrochemical activation. Enzyme immobilization was carried out either by a covalent carbodiimide coupling reaction to form amide bonds [64], or through simple physical adsorption. In a similar approach, Muñoz and co-workers [40] immobilized glucose oxidase via drop-casting and glutaraldehyde crosslinking on the surface of the pretreated electrode. Goel and co-workers [53,54] applied similar procedures for fabricating bioelectrodes used in biofuel cells. 

### 2.2. Direct Ink Writing

**Figure 4:**
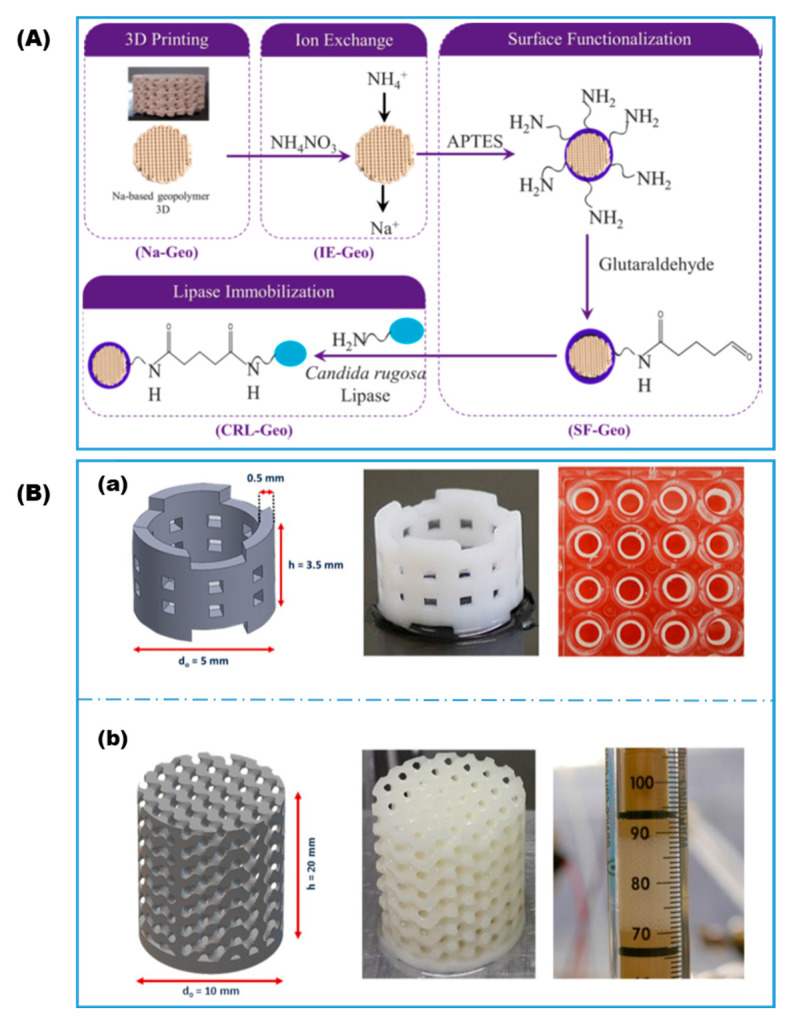
(**A**) Protocols for immobilizing lipase on 3D printed geopolymer lattices (Reproduced with permission from Ref. [58]). (**B**) Porous monoliths reaction beds for bioprocess engineering: (**a**) hollow cylinder that fits in the wells of a 96 multi-well plate for batch testing and (**b**) gyroid scaffold that fits in reactor column for dynamic testing (reproduced with permission from Ref. [60]).

Though less common than its use for enzyme entrapment, material extrusion by DIW is certainly compatible with post-printing enzyme immobilization. DIW can be used to print essentially any material as long as the precursor ink is formulated to possess favorable rheological behavior [33]. Dong et al. [57] printed silver nanoparticles directly onto a printed circuit board (PCB), which was later coated with a Nafion layer to assist the co-immobilization of lactate oxidase in a bovine serum albumin matrix for the construction of an electrochemical lactate biosensor. Dos Santos et al. [58] printed an aqueous paste of geopolymer precursor into a stacked lattice reactor packing followed by heat treatment to complete the geopolymerization. The fully cured inorganic 3D lattice was then functionalized through a series of ion exchange, amination, and glutaraldehyde activation (Figure 4A). Finally, lipase was covalently immobilized on the reactor lattice surfaces through the formation of Schiff’s base linkages. A high 91% of the initial activity was retained after the first reuse. 

### 2.3. Vat Photopolymerization with Digital Light Processing

Owing to its high resolution, vat photopolymerization with DLP has been used to print ultra-fine and complex structures for post-printing enzyme immobilization. Liu et al. [59] developed a continuous glucose sensing device using a DLP printed microneedle array as the support for subsequent conductive metal deposition and glucose oxidase drop-casting immobilization. The microneedles were able to penetrate the dermis layer of mouse skin to about 500 μm deep and responded accurately in real-time to the subcutaneous glucose levels modulated by food intake or insulin injection. Another advantage of DLP is the availability of wide selections of photosensitive monomers and crosslinkers that can be leveraged to create desired morphology and surface chemistries. Dimartino et al. [60] explored a suite of formulations including bifunctional acrylate monomers, diacrylate and tetraacrylate crosslinkers, as well as fatty alcohol porogens, for the fabrication of ordered porous beds for diverse chromatography and bioreactor applications (Figure 4B). In recent years, metal organic frameworks (MOFs) have been widely used for enzyme immobilization [65]. Limitations on making macroscopic structures with MOFs can be overcome with 3D printing. Doherty and co-workers [61] 3D printed a static mixer using DLP and functionalized it with a bovine serum albumin (BSA) coating followed by zeolitic imidazolate framework (ZIF) growth in the presence of organophosphate degrading enzyme A (OpdA) and a ZIF precursor mixture in a modified layer-by-layer fashion. The porous ZIF coating securely encapsulated enzymes on the surface of the in-line flow mixer while allowing for the free diffusion of reactants in and out of the ZIF cages.

### 2.4. Vat Photopolymerization by Stereolithography

Similarly, vat photopolymerization by SLA can be used to fabricate structured reaction beds out of various materials. An interesting example is the printing of a ceramic-based structured insert for enzyme immobilization in a continuous flow bioreactor by Valotta et al. [62]. In this process, the printing vat contains a mixture of alumina and photosensitive resin plus initiator, to serve as the ceramic precursor and binder, respectively. The preformed structure was thermally treated for debinding and sintering and the ceramic surface was easily functionalized using commercially available silanization reagents, such as (3-aminopropyl)triethoxysilane (APTES), for amination. Once amino groups are present on the surface, choices are available to proceed with either glutaraldehyde activation or carbodiimide activated ester reaction routes. Instead of using surface covalent immobilization, Razmjou and co-workers [48] grew ZIF encapsulated with carbonic anhydrase and formate dehydrogenase in tandem on the surface of SLA printed micromixers for the production of formic acid from CO_2_. These advances are expected to inspire more hybrid system innovations, taking advantage of each technology’s strength. 

## 3. Immobilization by Physical Entrapment during 3D Printing

Three-dimensional printing in the presence of enzymes requires holistic consideration of the whole process. In most cases, enzymes are not compatible with the high melting temperature used in FDM, whereas they readily tolerate aqueous or hydrogel-based systems, which resemble the environment where most natural enzymatic reactions take place. For that reason, with only a few exceptions, the entire 3D printing repertoire of enzyme immobilization by entrapment consists almost exclusively of hydrogel-based 3D printing, with DIW being the method most commonly employed. A list of recent studies is summarized in Table 3. 

### 3.1. Gel Printing

**Figure 5:**
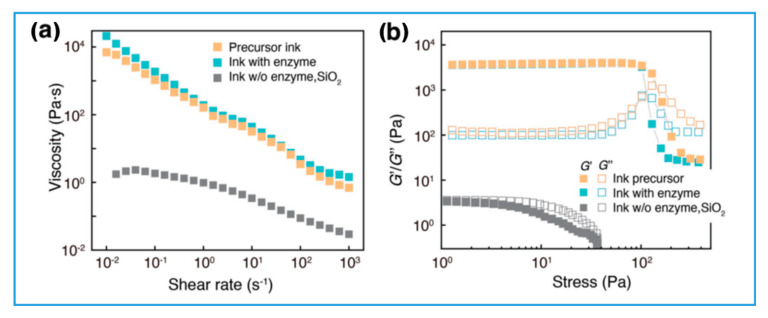
Evaluation of printability of hydrogel inks: (**a**) apparent viscosity vs. shear rate and (**b**) storage moduli (G′) and loss moduli (G″) vs. shear stress measured at oscillatory frequency of 1 Hz (reproduced with permission from Ref. [74]).

As previously mentioned, material extrusion by DIW is able to print practically any material type as long as proper rheology requirements are met with “ink” formulation [33]. A fundamental operational requirement for DIW is the ability to extrude a continuous, coherent filament which, upon deposition, is able to support its own weight and preserve the printed structure. On the other hand, the viscosity of the “ink” should not be so high that it impedes the extrusion or clogs the nozzle. In view of these requirements, shear-thinning, which is a non-Newtonian fluid behavior where viscosity decreases with increasing shear rate, is an essential characteristic for achieving printability with hydrogels. There are several common ways to formulate the ink for reaching desirable rheological properties. These include partial gelation through weak physical crosslinking [70,71,72], addition of colloidal clay particles [19,24], emulsification [44], partial gelation by enzymatic crosslinking [43,73], introduction of polymer entanglements [23,69] and addition of fused silica nanoparticles [74]. As shown in Figure 5a, including silica nanoparticles significantly increased the apparent ink viscosity while adding enzyme into the mixture did not affect the overall shear thinning behavior of the hydrogel ink. In addition, as shown in Figure 5b, the hydrogel ink with silica nanoparticles exhibited an elastic behavior at low shear stress (G′ > G″) and a prominent drop in storage modulus beyond a well-defined yield stress, at which point storage modulus crossed over the loss modulus. This shear thinning behavior is beneficial for the 3D printing process as it improves the flow of the ink through the nozzle. Then, after exiting the nozzle, the ink reverts back to an elastic self-supported gel state for better preservation of the structure. On the other hand, in the formulation without silica nanoparticles, storage and loss modulus overlap (G′~G″) and the ink is a free-flowing fluid without structural integrity. 

#### 3.1.1. Gelation by Physical Crosslinking

**Figure 6:**
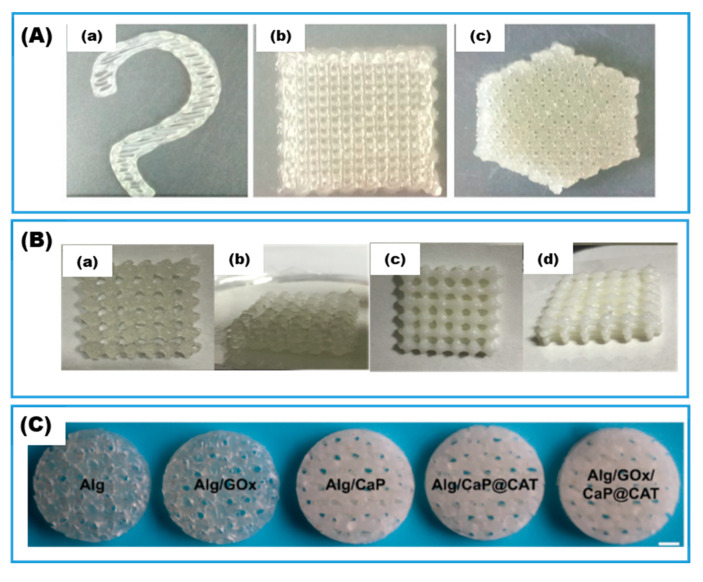
Alginate-based hydrogels. (**A**) The 3D printed xylanase entrapped in sodium alginate hydrogel: (**a**) hook-shaped; (**b**) quadrilateral lattice; (**c**) hexagonal lattice (adapted with permission from Ref. [70]). (**B**) Interpenetrating polymer network (IPN) and hybrid interpenetrating polymer network (HIPN) hydrogel: (**a**,**b**) sodium alginate/polyacrylamide (SA/PAm) IPN; (**c**,**d**) sodium alginate/polyacrylamide/hydroxyapatite (SA/PAm/HA) HIPN (reproduced with permission from Ref. [23]). (**C**) Appearance of 3D printed alginate-based hydrogel (Alg) with or without immobilized glucose oxidase (GOx), calcium phosphate micro-sheet (CaP), or catalase-induced biomineralized calcium phosphate nanosheet (CaP@CAT) (adapted with permission from Ref. [72]).

When natural polyelectrolytes such as alginate (−) and chitosan (+) are used as a component of the ink, it is customary to crosslink them through weak electrostatic attractions, with each other or with charged chelators such as Ca^2+^ for alginate and ethylenediaminetetraacetic acid (EDTA) for chitosan. Additional crosslinking with the same or different mechanism is usually required to strengthen the bonding and improve the mechanical properties of the printed hydrogel. For example, Jiang and co-worker [70,71] first mixed CaCl_2_ solution with sodium alginate solution to achieve printable shear-thinning ink followed by an additional crosslinking step where the printed object was immersed in the CaCl_2_ solution to strengthen the mechanical properties (Figure 6A). Alternatively, Cao and co-workers [23,69] introduced a second acrylamide/bis-acrylamide system that was covalently crosslinked through chemical initiation using ammonium persulfate (APS) and tetramethylethylenediamine (TEMED) (Figure 6B). Because the chemical initiation began instantaneously once the initiators were mixed in, the prepared ink had to be kept cold, (i.e., stored in a refrigerator) to slow down the chemical crosslinking and extend the printable time window. The addition of inorganic hydroxyapatite resulted in a hybrid interpenetrating polymer network (HIPN) hydrogel that had more than 50 times higher compression modulus compared to a sodium alginate hydrogel. Remarkably, the 3D-printed enzyme immobilized hydrogel achieved 100% protein recovery and retained 95% of enzyme activity after 35 days at 4 °C. When glucose oxidase and catalase were co-immobilized in the HIPN hydrogel with different macro-pore densities as prescribed by 3D CAD designs, the gluconic acid production rate in a bioreactor at 35 °C and pH 4.0 increased with higher macropore density, indicating a mass transfer benefit of producing finer 3D structures. A number of hydrogels with high macro-pore densities outperformed free enzymes in the bioreactor. The authors attributed this improvement to the close proximity of the location of the two enzymes immobilized in the hydrogel compared to the larger average distance between free enzyme molecules in the liquid. The concept of enzyme cascade and co-immobilization is already widely recognized [63] as one of the most promising directions for designing more efficient biocatalysis processes. Yang et al. [72] studied the size effect of inorganic reinforcements on the compressive strength of enzyme-immobilized alginate-based hydrogels (Figure 6C). While adding calcium phosphate micro-sheets improved the compressive strength, using lab-made biomineralized calcium phosphate nanosheets performed even better. Meanwhile, adding glucose oxidase enzyme into either the non-reinforced or reinforced hydrogels did not alter their mechanical properties. Notably, the biomineralized nanosheet contained catalase entrapped during its synthesis which works in concert with glucose oxidase by removing its reaction product H_2_O_2_ in a cascade reaction and raises the overall glucose removal rate of the hydrogel. Recently, Shavandi and co-worker [77] functionalized both chitosan and alginate with phenol side groups and printed the double-crosslinked chitosan alginate hydrogel (DCCA) for biomedical applications. The first crosslinking mechanism was through the electrostatic attractions between the positively charged chitosan with negatively charged alginate forming a polyelectrolyte complex while the second mechanism was by covalent bonding through an enzyme-mediated formation and recoupling of α-carbon and or phenoxy radicals using horseradish peroxidase and H_2_O_2_. The rate of crosslinking was controlled by adjusting H_2_O_2_ concentration, allowing optimization of a printable time window. DCCA exhibits micro-fibrous morphology under SEM observations, which is distinctive from either chitosan or alginate hydrogel alone and could account for its higher toughness compared to two single-crosslinked hydrogels.

A special case of physical crosslinking is exemplified by 3D printing of thermoreversible agarose-based hydrogels. It was proposed that agarose transitions from random coil to rigid rod-like conformation when cooling below the transition temperature of 60 °C, stabilized by intramolecular hydrogen bonding. Further cooling below 40 °C promotes intermolecular hydrogen bonding and the eventual macroscopic sol–gel transition [83]. Rabe and co-worker [39,66] leveraged these thermoreversible hydrogen bonding interactions by heating the print head and cooling the substrate platform, to break and reform the physical crosslinks, respectively, for a successful DIW printing process. The agarose-based system technically is able to immobilize all types of enzymes, though it is more suitable for thermally stable enzymes that can tolerate the brief heating at 60 °C. Nevertheless, a wide selection of enzymes can be immobilized using this hydrogel-based DIW method in comparison to the usual ≥100 °C print head temperature for FDM. Immobilized enzymes exhibited better solvent resistance and good catalytic performance in a stackable modular continuous flow reactor. However, enzyme leaching was more severe with lower molecular weight enzymes versus higher molecular weight enzymes. Further research on controlling the pore size of the hydrogel matrix could potentially improve the working longevity of this thermoreversible gel enzyme immobilizing system.

#### 3.1.2. Gelation by Photo-Crosslinking

**Figure 7:**
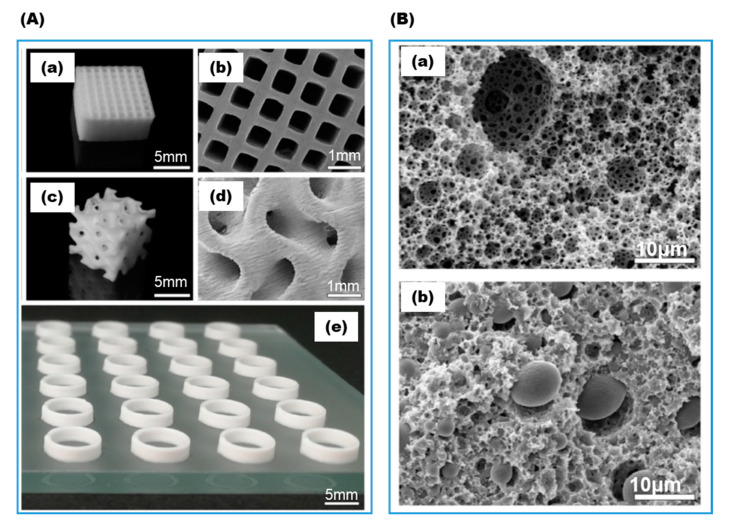
Hydrogel-filled high internal phase emulsion (HIPE). (**A**) The 3D printed scaffold: (**a**,**b**) cuboid grid structure with an edge length of 10mm and a height of 5 mm; (**c**,**d**) cubic gyroid structure with an edge length of 8mm; (**e**) hollow cylinders as used for activity assays. (**B**) Cross sectional morphology of HIPE: (**a**) no monomer in aqueous phase with empty voids and interconnecting pores between the voids; (**b**) with monomers in the aqueous phase forming hydrogel filling up the voids (adapted with permission from Ref. [44]).

Many photo-sensitive resins can be cured rapidly and precisely where light lands on the material. When aqueous enzyme solutions are present, photo-crosslinked hydrogels with entrapped enzymes are formed. However, because resins are usually low molecular weight monomers or macromers, they have relatively low viscosity unsuitable for extrusion-based 3D printing, i.e., DIW, where dripping could occur. To circumvent this issue, viscosity enhancers, such as colloidal particles and high molecular weight polymers, are formulated into the ink to achieve desirable rheological properties. Franzreb and co-worker [19,24] tested two types of colloidal clay particles and a branched polysaccharide for modifying the rheology of PEG-DA resin and found that the smaller clay particle resulted in stronger shear-thinning behavior while the larger clay particle and the branched polysaccharide polymers yielded higher dynamic viscosities at higher shear rate. The immobilized enzymes exhibited low activity recoveries or effectiveness factors of only about 10% compared to the same concentration of free enzymes, owing to the slowed substrate and product diffusion processes within the hydrogel relative to the apparent rate of the reaction which the enzyme catalyzed. In fact, mass transfer limitations are more evident with faster acting enzymes, resulting in lower effectiveness factors. This hypothesis was confirmed by the corresponding multifold increase in reaction rate with increase in the surface areas of the fragmented hydrogels. This example demonstrates the need to develop 3D printed hydrogels with smaller and finer structures that could result in substantial improvement of the catalytic performance. To this point, Hubbuch and co-workers [44] demonstrated that a significantly shorter equilibration time was achieved in their hydrogel-filled high internal phase emulsion (HIPE) scaffold when a smaller inner diameter nozzle was used to reduce the overall thickness of the printed cylinder shaped samples, thus reducing the reaction path length. In addition, increasing surfactant and aqueous phase mass fraction also helped with alleviating mass transfer limitation by increasing degree of openness and reducing the hydrogel droplet sizes (Figure 7).

**Figure 8:**
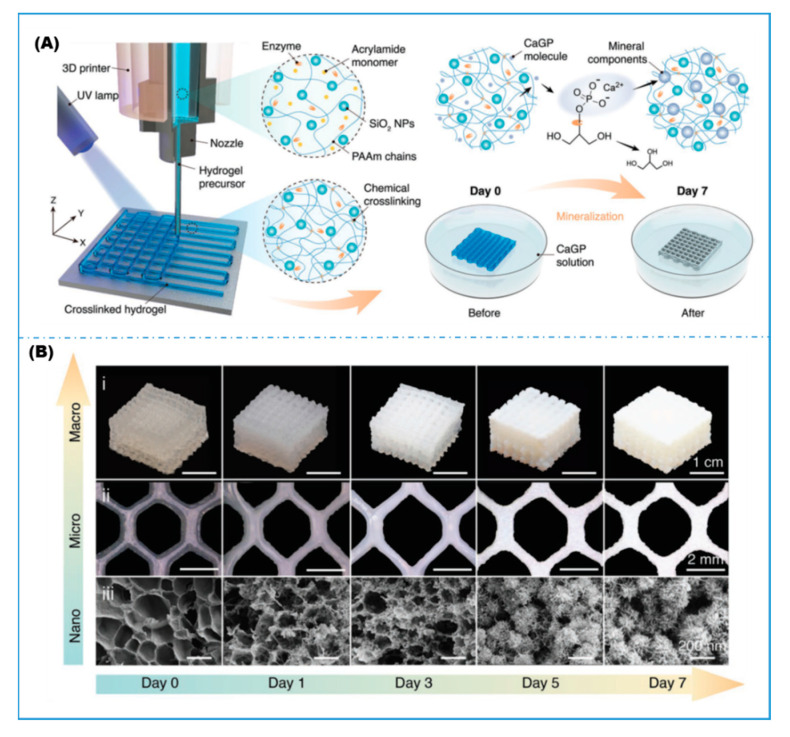
Biomineralized 3D printed hydrogel. (**A**) Schematic protocols for printing enzyme-loaded hydrogel precursors and enzyme-induced biomineralization. (**B**) Multi-scale observations of the mineral growth in hydrogel (reproduced with permission from Ref. [74]).

A common challenge facing photo-crosslinked hydrogels, or all hydrogels in general, is their weak mechanical properties. Chen et al. [74] used DIW coupled with UV curing to print photo-crosslinkable acrylamide-based resin loaded with alkaline phosphatase (ALP). The printed hydrogel lattices were then immersed in calcium glycerophosphate (CaGP) solution for biomineralization over a period of 7 days. The immobilized ALP enzyme catalyzes the dephosphorylation of the CaGP which resulted in the formation of calcium phosphate nanoparticles within the hydrogel (Figure 8). Both hardness and reduced modulus obtained by nanoindentation testing showed increases by 3–4 orders of magnitude, attributable to the formation of a percolated mineralized structure. Dog-bone shaped tensile testing samples were also 3D printed both longitudinally and transversely. Biomineralization enhanced the tensile moduli by more than 1000 times and tensile strength by more than 30 times irrespective of printing path direction. Compression testing confirmed a more than 10-fold increase in peak compression stress due to biomineralization. Through this example, we can foresee a rapid growth in the area of combining 3D printing technology with immobilized-enzyme-induced biomineralization and biocatalysis for fabricating hierarchically ordered functional composite materials. 

#### 3.1.3. Gelation by Enzymatic Crosslinking

Covalent crosslinking catalyzed by enzymes is gaining attention as a safer alternative to the use of chemical crosslinking agents. Gelatin, a biocompatible bioink for 3D printing, could benefit from mild covalent crosslinking because it suffers from thermally unstable physical gelation and inherent low viscosity [45]. Physically crosslinked gelatin hydrogel melts at physiological temperature while its enzymatically crosslinked counterpart retains full structural features under the same condition. The enzyme transglutaminase works by catalyzing the formation of isopeptide bonds between side chains of glutamine and lysine. By adding 0.65 wt% bacterial cellulose as a viscosity enhancer, Zhou et al. [45] were able to 3D print objects with different shapes and complexity, including ear, meniscus, bowl, and 3D lattice. Because the enzyme was added before 3D printing, its dose and time responses become critical to prevent over-crosslinking and maintain printability of the bioink. Hashimoto and co-worker [43] extended the printability window by preheating gelatin for long hours, before mixing in transglutaminase, which works by essentially reducing the molecular weight of the gelatin precursor. Zhou et al. [73], on the other hand, deactivated transglutaminase as soon as a sufficient viscosity of the gelatin methacryloyl (GelMA) was reached and fully cured the hydrogel through a post-printing photo-crosslinking process.

**Figure 9:**
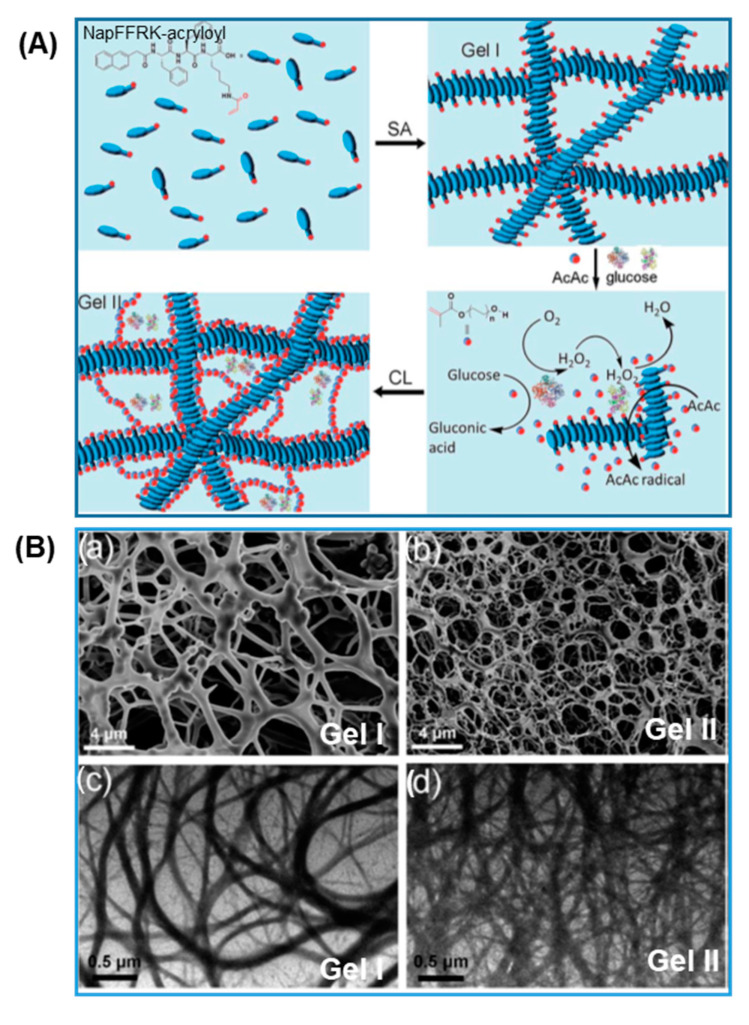
Dual-enzyme catalyzed gelation of NapFFRK-acryloyl based hydrogel. (**A**) Schematics of the formation of pH regulated self-assembly of supramolecular hydrogel (Gel I) and radical polymerized and crosslinked hydrogel (Gel II). (**B**) SEM images of: (**a**) Gel I and (**b**) Gel II; TEM images of: (**c**) Gel I and (**d**) Gel II (adapted with permission from Ref. [68]).

A second enzyme-induced crosslinking mechanism relies on the radical forming oxidation activity of horseradish peroxidase (HRP) in the presence of H_2_O_2_, which can be exogenously supplied or generated in situ by other reactions such as the oxidation of β-D-glucose catalyzed by glucose oxidase (GOx) in the presence of oxygen and flavin adenine dinucleotide (FAD) cofactor. Wang and co-workers [67,68] utilized the pH lowering effect of the GOx catalyzed glucose oxidation reaction, which produces gluconic acid, to induce the self-assembly of guanidinium-containing oligopeptide (NapFFRK-acryloyl) into a supramolecular hydrogel. The generated H_2_O_2_ was then used by HRP for oxidizing acetyl acetone into its free radical for initiating the polymerization of poly(ethylene glycol) methacrylate (PEGMA) and crosslinking with the acryloyl groups on the self-assembled NapFFRK-acryloyl gel as illustrated in Figure 9A. The double-crosslinked Gel II exhibited smaller pore size and many more entangled nanofibers compared to Gel I. (Figure 9Ba–d) As a result, Gel II had a storage modulus 16.4 times that of Gel I and was able to stretch 2.5 times its initial length without breaking. The self-healing properties were illustrated in their study by bending a healed joint without breaking. Thus, the dual-enzyme crosslinked hydrogel was selected for 3D printing, showing promise in the fields of self-healing [84] and hemostatic biomedical applications.

**Figure 10:**
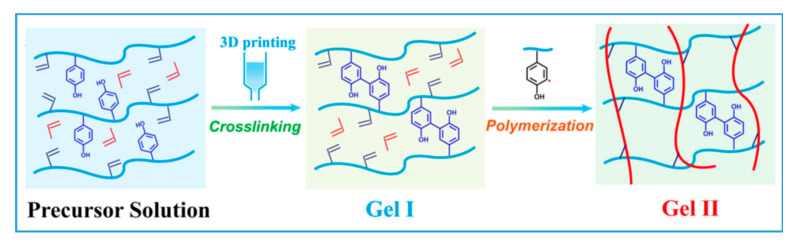
Schematics of enzyme-catalyzed dityrosine bond formation used for 3D printing. Glycidyl methacrylate (GMA) and tyrosine grafted chondroitin sulfate hydrogel (reproduced with permission from Ref. [76]).

Recently, Wang’s research group [76] employed the same set of GOx/HRP enzymes on the dual-crosslinking of glycidyl methacrylate (GMA) and tyrosine grafted chondroitin sulfate, a polysaccharide, for use as bioink for 3D printing (Figure 10). The formation and recoupling of α-carbon radicals to form dityrosine linkages was found to be rapid, as evidenced by the absence of carbon radicals by electron paramagnetic resonance (EPR) measurement within the initial 30 min, whereas radicals appeared 3 h after mixing, indicating the bioink’s ability to initiate the polymerization of the monomer gradually, extending the printable time window. Through the same mechanism, Burke and co-workers [75] leveraged the natural tyrosine content in silk fibroin protein and its crosslinking catalyzed by HRP with the addition of exogenous H_2_O_2_, the dose of which can be used to fine tune the rheological properties and printable time window of the bioink. The H_2_O_2_ concentration was initially kept low for the preparation of printing ink with a low G′, followed by a post-printing dose to achieve a fully crosslinked stable silk hydrogel.

### 3.2. Molten Matrix

The entrapment of enzymes in thermoplastic 3D printed structures is an exception rather than a common practice in the use of FDM technology. However, depending on the ultimate application goals, entrapping enzymes inside the thermoplastic matrix may be beneficial. Greene et al. [82] mixed solid-state lipase enzyme powder together with dry ground polycaprolactone (PCL) powder and melt-extruded the “thermal paste” in an FDM-like 3D printer. Dry solid enzymes were able to well tolerate the 130 °C processing temperature for 60 min. The embedded lipase accelerated the degradation of PCL when immersed in buffer solution compared to using the same amount of dissolved free enzyme. The faster degradation was attributed to the fact that dissolved lipase has to first adsorb onto the surface of the PCL film and work its way in whereas the embedded lipase initiates the degradation as soon as water diffuses into the film through pores caused by the presence of solid enzyme powder in the printing paste. This special case demonstrated that although enzymes usually have low thermal stability in their hydrated state, solid state enzymes can tolerate much higher processing temperatures, which permits melt-based 3D printing techniques for special applications.

### 3.3. Low Viscosity Resin

**Figure 11:**
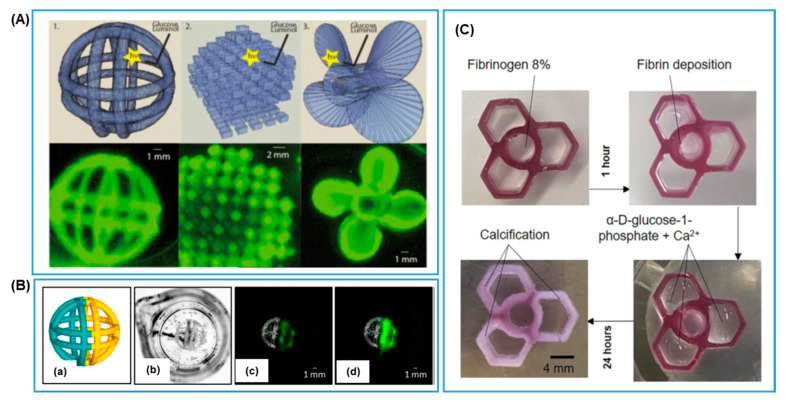
DLP 3D printed PEG-DA based enzyme immobilized hydrogel. (**A**) CAD drawings of complex 3D hydrogel objects entrapping both horseradish peroxidase and glucose oxidase and their chemiluminescent images in the presence of glucose and luminol (reproduced with permission from Ref. [78]). (**B**) Bi-enzyme heterogeneous system: (**a**) CAD drawing of a heterogeneous fanciful ball composed of glucose oxidase (**left**) and peroxidase (**right**); (**b**) visible light image of the printed heterogeneous fanciful ball, and chemiluminescent image of the homogeneous fanciful ball in the presence of glucose and luminol at (**c**) 60 and (**d**) 90 min after substrates addition (From Ref. [47] 2017 MDPI). (**C**) Sequential catalytic activities of thrombin and alkaline phosphatase leading to fibrin deposition and calcification (Reproduced with permission from Ref. [79]).

VP, including both DLP and SLA, uses photo-curable resins that are also common for the light-based DIW, but differs from DIW in that it does not require high viscosity. Enzymes can be mixed in the aqueous ink formulation which pervades throughout the whole resin vat. For this reason, the total amount of enzyme needed to print a hydrogel object with certain volumetric concentration will depend on the size of the vat and the waste increases as the ratio of vat working volume to printed object volume increases. Nevertheless, owing to its working mechanism through the manipulation of light path or pattern, complex structures that are hard to print using DIW are made possible using VP. Marquette and co-workers [78] combined horseradish peroxidase and glucose oxidase with PEG-DA macromer and DLP 3D printed it into multiple complex structures, such as a fanciful ball, 3D pixel and propeller. (Figure 11A) The co-immobilized enzyme pair GOx/HRP worked in concert and illuminated the whole structure in the presence of the substrate glucose and chemiluminescent reagent luminol. In addition, spatial heterogeneous immobilization was explored by separating the two enzymes in two parts of the printed structures by dissolving enzymes in two separate resin batches, then pausing and switching the resin in the middle of the 3D printing process [47]. As shown in Figure 11B, only the part of the fanciful ball immobilized with HRP became illuminated and the light intensity increased over time. This can be explained by the diffusion of H_2_O_2_ into the part where HRP resides to complete the full reaction cascade that eventually emits light. In a later study, co-immobilized thrombin and alkaline phosphatase on a 3D printed hydrogel successfully induced fibrin deposition and calcification (Figure 11C). In a recent study, Basit and co-workers [46] immobilized laccase on torus-shaped PEG-DA hydrogels that were printed using SLA. The immobilized laccase showed high thermostability and pH tolerance and was able to effectively remove 95% of diclofenac and ethinylestradiol from the aqueous solution within 24 and 2 h, respectively. 

### 3.4. Novel Variations

**Figure 12:**
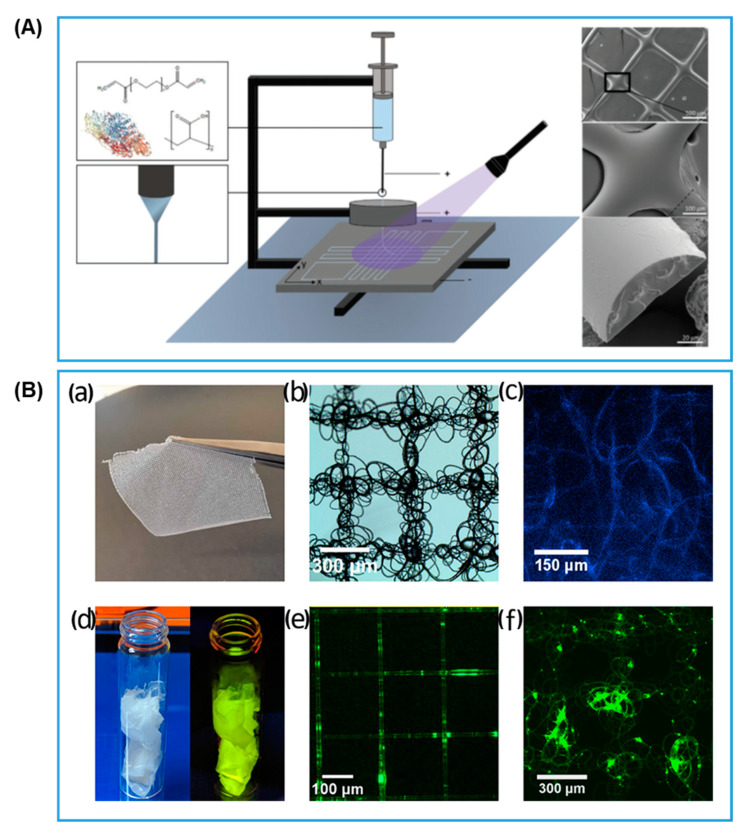
(**A**) The 3D jet writing of hydrogel fibers yielding oriented hydrogel fibers loaded with enzymes (reproduced with permission from Ref. [80]). (**B**) Melt electrowriting of PCL with entrapped electrostatically stabilized enzymes/proteins: (**a**) photo of the fabric with area of 3 cm^2^; (**b**) widefield microscopy view of the fine structures of the printed fabric with fiber thickness <5 μm; (**c**) enzymatic activity after the melt electrowriting process as shown by the fluorescent emission from the enzyme catalyzed hydrolysis reaction; (**d**) fluorescent plastics created by entrapping superfolder green fluorescent protein into PCL; (**e**) printed ordered grid structure with fluorescent PCL; and (**f**) the fluorescent plastics were used to print fabrics with repetitive tangles (reproduced with permission from Ref. [81]).

As already mentioned, there has been a need for developing 3D printed hydrogels with smaller and finer structures to reduce mass transfer limitations and improve the effectiveness factor of entrapped fast-acting enzymes [85]. Lahann and co-workers [80] developed a new 3D printing technique named “3D jet writing” to create micro-sized polymer hydrogel fibers oriented in a self-supporting 3D scaffold. At a glance, the equipment set-up looks like a combination of DIW and electrospinning, with a print head nozzle attached to a high voltage power supply and the platform grounded. The addition of a metal ring structure in between the tip of the nozzle and the grounded platform provides an intermediate voltage to stabilize the polymer jet against bending and whipping, which is essential for electrospinning to generate finer fibers, but leads to random deposition of the fibers (Figure 12A). Through a parallel control experiment, the authors found that 3D jet writing of PEG-DA/PAA was able to produce fibers 6.5 times thinner than conventional DIW. The entrapped β-galactosidase showed a much-improved effectiveness factor of 21.2% and retained a high level of activity after 3 days of continuously flowing reactor operation. 

Examples of novel technological variations also exist for non-aqueous systems. Perriman and co-workers [81] stabilized enzymes electrostatically through sequential addition of a pair of surfactants to the enzyme solution and retrieved organic solvent-soluble and melt-processable solid stabilized enzymes through dialysis and lyophilization. Then, they went on to use this stabilized enzyme in solvent casting, organic solvent-based solution extrusion, thermal molding, and, most interestingly, melt electrowriting. As shown in Figure 12B, fine fibers were printed in a well-defined pattern and formed a fabric structure that can be handled conveniently and retained all of its original enzyme activity. This fabric-forming feature could be useful for future scale-up of immobilized enzymes for large scale industrial applications. Another example using organic solvent systems involves the synthesis of random heteropolymers (RHP) with co-monomer distribution matching the protein surface pattern of the enzyme for stabilization and solubilization in organic solvents [86]. Xu and co-workers dissolved RHP-stabilized lipase together with PCL and a conductive filler for DIW of biodegradable electronic circuits [87]. The printed circuit remained functional and fully degradable, upon immersion in warm water, for at least 7 months at room temperature storage and after 1-month continuous exposure to electrical voltage. After enzyme facilitated degradation of PCL is carried out, the metal conductive fillers can then be recovered and recycled. 

## 4. Limitation of the Review

The scope of this review focused only on studies that directly immobilized enzymes on or in the 3D printed objects and does not include many other innovations that use 3D printing to make customizable tools and devices, such as labware [88], mass spectrometry microcolumns [89], and biomedical devices [90], where enzymes are not directly included. The review emphasized the most recent techniques and chemistry for achieving successful enzyme immobilization by 3D printing, especially by gelation mechanisms that favor high retention of enzyme activity and longevity; however, the reader is referred elsewhere for more information on the basic physics of mass transfer limitations that may occur in these systems and how they are currently being studied through modeling [85]. 

## 5. Conclusions and Future Perspectives

Three-dimensional printing has long been explored together with whole-cell systems. Then, following a few early publications in 2016, the number of research reports on systems where cell-free enzymes are immobilized in conjunction with 3D printing has continued to grow. The application areas have expanded from initial biosensing and tissue engineering applications to now include many biocatalytic reactor devices for selective synthesis, degradation, and transformation of chemicals and toxins. Still, the total body of work is limited and only a handful of common entry-level 3D printing methods are being routinely used, namely FDM, DIW, SLA and DLP. Most studies to date are proof-of-concept in nature. Nevertheless, these early boundary-spanning studies have already identified exciting new potentials as well as a number of challenges that require future investigation. 

The success of 3D printed immobilized enzymes for biocatalysis hinges heavily on minimizing diffusion limitations that limit catalytic rates, which could partially be overcome by improving the printing resolution of popular economical techniques, namely hydrogel-based direct ink writing. There are already a number of developments, such as 3D jet writing [80] and melt electrowriting [81] for creating micro-sized enzyme-entrapped polymer fibers with well-defined 3D patterns with fine structural features to mitigate diffusion limitations and aid mixing. General progress in 3D printing technology development, i.e., higher resolution and speed, and lowering of the cost of the most advanced 3D printing technologies will also provide advances for enzyme immobilization. For example, continuous liquid interface production (CLIP) will allow for up to 1000 times faster printing rates than conventional DLP, and multi-jet printing (MJP) could make it possible to print objects composed of multiple materials, potentially entrapping multiple enzymes in spatially well-defined complex structures at the same time with a 16 μm resolution [91]. Layer-by-layer printing has been the paradigm for most of the 3D printing methods, until recently, when a layerless light controlled method called volumetric bioprinting has emerged [92]. This method enables hydrogel printing at fast rates with high precision and overcomes the technical difficulties of printing unsupported structures, such as overhangs. Applications in printing biomimetic organoids have shown success [93] and adaptation to enzyme immobilization is likely to happen soon. Meanwhile, rapid biotechnology developments are making it easier to modify native enzymes to include beneficial binding domains [31] that interact or bond to the 3D printed surfaces spontaneously [94]. This will allow future development of reusable 3D printed devices that can be re-immobilized with enzymes after initial activity loss with a single dip of the 3D structure or create highly targeted constructs by VP techniques with minimal loss of enzymes in the vat phase.

Currently, most multi-enzyme cascades are created either by post-printing multi-step enzyme immobilization combined with masking procedures for patterning or through the change of printing ink or resins while the printer is paused; both are laborious procedures. Imminent solutions to this problem are to increase accessibility of the aforementioned MJP and accelerate the adoption of other multi-material printing techniques, such as the use of core-shell co-axial nozzles or multi-nozzle arrays in DIW [33]. We expect these, and even more advanced techniques, to promote extensive future exploration of biomimetic enzyme cascades that employ diverse enzyme selections—such cascades are the essence of life. With disparate materials arranged in an optimized pattern and bioengineered enzymes binding only to specific target materials, enzyme cascades will become commonplace. Other strategies originating from innovations in conventional enzyme immobilization techniques will also be utilized. For example, MOFs were self-assembled in-situ on the surface of a 3D printed micromixer while simultaneously encapsulating enzymes [48]. Alternatively, we imagine that in the near future enzyme-encapsulated MOFs could be conveniently dispersed in any 3D printing matrix forming hybrid enzyme immobilization techniques. Natural and synthetic nanoparticles as well as 2D (sheets) and 1D (nanotubes) carbonaceous materials could also act as hybrid enzyme-carriers in 3D printed matrices. Because enzymes catalyze reactions over time, the concept of 4D printing was introduced to the enzyme immobilization field by Marquette and colleagues [47] to anticipate the ability of 3D printed objects containing enzymes “to evolve over time and under external stimulus by modifying their shape, properties or composition.” Time-dependent functionality could incorporate multiple enzyme activities for structure fabrication and stimuli responsiveness, such as biomineralization, self-healing, and autonomous shape-changing [95]. Multiple functionalities come with unlimited possible combinations and high innovation potential for future work.

Importantly, though our discussion was confined to studies with enzymes directly immobilized on or inside the 3D printed structure, further progress in this convergence field is not separable from the independently evolving fields of 3D printing [16], conventional enzyme immobilization [65], 3D printed microfluidic devices [96], and bioprinting [35]. Extensive literature exists on employing 3D printing methods in biomedical engineering field [97] for live cell cultures and delivering bioactive molecules. With more research, materials and methods developed in these areas can be translated to immobilizing cell-free enzymes. For example, many polysaccharide-based hydrogel materials, such as hyaluronic acid, pectin, and various cellulose derivatives, which have already been widely used for drug delivery [98] and tissue engineering [99,100] could be applied to cell-free systems. In particular, many bacterial polysaccharides have been shown recently to exhibit antibiofilm activity [101] and the possibility of utilizing this bioactivity in 3D printed immobilized enzyme seems extremely promising for preventing biofouling and improving enzyme longevity.

Looking forward, technological advances in 3D printing with hydrogel materials will allow us to embark in rudimentary ways on creating structures and linking reaction cascades following a path that nature has perfected over billions of years in living cells; packing thousands of different types of enzymes into compartmentalized spaces mostly filled with gel-like fluids that carry out complex chemical reactions efficiently and cooperatively in response to biological stimuli. Rapid development across the spectrum of 3D printing techniques brings us closer to eventually fully understanding and emulating the biological world that we live in.

## Figures and Tables

**Table 1:** Comparison of biocatalysts fabricated by different immobilization methods.

Method	Free Enzyme	Conventional Immobilization	3D Printed Immobilization	
			Post-Printing	Entrapment
Advantages	Ready-to-use; high selectivity; easily mixed; high mass transfer; mild operational conditions	High stability; reusable; some continuous flow geometries; large variety of material options; constant innovation in the field	High stability; reusable; continuous flow; customizable complex structure; scalable and low material waste; applicable for all 3D printing methods; wide variety of printable materials	High resolution; precise enzyme placement; high stability; reusable; continuous flow; customizable complex structure; scalable and low material waste; single step fabrication; mild immobilization conditions; versatile for multi-enzyme assemblies or cascades
Challenges	Low stability; frequent replenishment; non-recyclable	Difficult to form complex structures; may require separate recycling steps; mass transfer limits; harsh chemicals; limited mechanical properties	Harsh chemicals for surface modification; time-consuming multi-step processes; difficult for enzyme cascade	Limited material types; limited mechanical properties; mass transfer limits
Future development	Protein engineering for stabilization and surface functionalization	More sustainable materials; milder chemical reactions; more efficient geometries	More sustainable materials; milder chemical reactions; combine advancements across conventional enzyme immobilization and 3D printing fields	New bioink materials; new mechanical reinforcement methods; highly controlled geometries; ultra-high (nano-scale) resolution

**Table 2:** Techniques for post-printing enzyme immobilization.

3D Printing Technique	Immobilization Mechanism	Surface Materials	Reagents	Enzymes	Application	Name, Year, and Ref.
FDM	Covalent attachment; drop-casting	ABS	GA	GOx; LOx	Biosensing	Su and Sun, 2016, 2018 [20,22]
	Covalent attachment	Nylon	GA	ω-transaminase	Biocatalytic reactors	Sans, 2017 [37]
	Physical adsorption; covalent attachment	Graphene/PLA; Carbon black/PLA	EDC/NHS	ALP; HRP; GOx; L-amino acid oxidase	Biosensing	Pumera, 2019–2021 [41,50,51,52]
	Drop-casting	Graphene/PLA	GA	GOx	Biosensing	Muñoz, 2020 [40]
	Physical adsorption; covalent attachment	Carbon/PLA	EDC/NHS	GOx; Laccase	Biofuel cell	Goel, 2020 [53,54]
	Physical adsorption	PLA	Aniline silane	Lipase	Biocatalytic reactors	Chu and He, 2021 [42]
	Covalent attachment	PLA	Chitosan coating with GA	Laccase	Biocatalytic reactors	Stamatis, 2022 [55]
	Covalent attachment; physical adsorption	Carbon-fiber reinforced PLA	Various silane coupling agents; GA	PGA; protease; glycosidase; lipase	Biocatalytic reactors	Gao and He, 2019 [56]
DIW	Drop-casting	Nafion	BSA	Lactate oxidase	Biosensing	Kim, 2018 [57]
	Covalent attachment	Na-based geopolymer	Aminosilane; GA	Lipase	Biocatalytic reactors	dos Santos, 2021 [58]
DLP	Drop-casting	Gold	GA	GOx	Biosensing	Yang and Cui, 2021 [59]
	Covalent attachment	Poly(2-carboxyethyl acrylate)	EDC	Trypsin	Biocatalytic reactor	Dimartino, 2022 [60]
	Encapsulation in ZIF grown on surface	ABS; BSA	ZIF precursor	OpdA	Wastewater remediation; Industrial biocatalysis	Doherty, 2020 [61]
SLA	Covalent attachment	Ceramic	Aminosilane; GA or EDC/NHS	Phenolic acid Decarboxylase	Biocatalytic reactors	Valotta and Gruber-Woelfler, 2021 [62]
	Encapsulation in ZIF grown on surface	PD/PEI	ZIF precursor	CA; FDH	Biocatalytic reactors	Razmjou, 2021 [48]
Polyjet	Covalent attachment	Poly(acrylic acid)	EDC	GOx; HRP	Biocatalytic reactors	Franzreb, 2016 [63]

Acronyms: FDM—fused deposition modeling; DIW—direct ink writing; DLP—digital light processing; SLA—stereolithography; ABS—acrylonitrile butadiene styrene; GA—glutaraldehyde; HCl—hydrochloric acid; PLA—poly(lactic acid); EDC—1-ethyl-3-(3-dimethylaminopropyl)carbodiimide; NHS—N-hydroxysuccinimide; BSA—bovine serum albumin (BSA); GOx—glucose oxidase; LOx—lactate oxidase; ALP—alkaline phosphatase; HRP—horseradish peroxidase; PGA—penicillin G acylase; ZIF—zeolitic imidazolate frameworks; OpdA—organophosphate degrading enzyme A; CA—carbonic anhydrase; FDH—formate dehydrogenase; PD—polydopamine; PEI—polyethyleneimine.

**Table 3:** Physical entrapment strategies for enzyme immobilization during 3D printing.

3D Printing Technique	Materials	Gelation Mechanism	Enzymes	Application	Name, Year, and Ref.
	PEG-DA hydrogel; Colloidal clay and branched polysaccharide as viscosity enhancer	Photo-crosslinking	ADH; BFD; β-Gal	Biocatalytic reactor	Franzreb, 2018, 2019 [19,24]
	Thermoreversible agarose-based hydrogel	Temperature-induced	Esterase; ADH; decarboxylase	Biocatalytic reactor cartridge	Rabe, 2018, 2019 [39,66]
	PAA/PEG-DA hydrogel spheres enclosed in hydrophobic acrylates matrix	Photo-crosslinking	β-Gal	Biocatalytic reactors; rapid screening of immobilization formulation	Hubbuch, 2020 [44]
	NapFFRK-acryloyl and PEG-MA hydrogel	Enzyme-triggered pH change and radical polymerization	GOx; HRP	3D cell culture; tissue engineering	Wang, 2016 [67,68]
	SA/PAm/hydroxyapatite hybrid interpenetrating polymer network (HIPN) hydrogel	Electrostatic; chain entanglement	GOx; CAT; Laccase	Biocatalytic reactors; wastewater remediation	Cui and Cao, 2019, 2020 [23,69]
DIW	SA hydrogel	Electrostatic	Xylanase; Aldo-keto reductase	Biocatalytic reactors	Jiang and Zhou, 2020, 2022 [70,71]
	SA hydrogel reinforced by calcium phosphate nanosheets	Electrostatic	GOx; CAT	Bone tissue engineering	Wang and Huang, 2021 [72]
	Gelatin-based hydrogel	Enzymatic crosslinking	TGase	Bioprinting; food printing	Hashimoto, 2020 [43]
	Gelatin-based hydrogel/bacterial cellulose	Enzymatic crosslinking	TGase	Bioprinting; tissue engineering	Hu and Wang, 2021 [45]
	Gelatin methacryloyl (GelMA) hydrogel	Enzymatic crosslinking; photo-crosslinking	TGase	Bioprinting; tissue engineering	Lee and Tan, 2019 [73]
	PAm hydrogel/fused silica nanoparticles	Photo-crosslinking	ALP	Biomineralization; biomedical	Liu, 2022 [74]
	Silk fibroin hydrogel	Enzymatic crosslinking (dityrosine bond)	HRP	Bioprinting; tissue engineering	Burke, 2020 [75]
	Polysaccharide derivative/PAm hydrogel	Enzymatic crosslinking (phenolic); enzyme-initiated radical polymerization	GOx; HRP	Bioprinting; tissue engineering	Wang, 2020 [76]
	Phenol functionalized chitosan and alginate hydrogel	Electrostatic; enzymatic crosslinking (Phenolic)	HRP	Bioprinting; tissue engineering	Shavandi, 2022 [77]
DLP	PEG-DA hydrogel	Photo-crosslinking	GOx/HRP; ALP; thrombin	Biosensing; tissue engineering; biomineralization	Marquette, 2016, 2017 [47,78,79]
SLA	PEG-DA hydrogel	Photo-crosslinking	Laccase	Water remediation	Goyanes and Basit, 2022 [46]
3DJW	PEG-DA hydrogel/PAA	Photo-crosslinking	β-Gal	Biocatalytic reactors	Lahann, 2020 [80]
MEW	PCL	N/A (Solidification)	PTE; sfGFP	Self- decontaminating surfaces; fully biodegradable fluorescent plastic	Perriman, 2021 [81]
FDM	PCL	N/A (Solidification)	Lipase	Biodegradable plastics	Greene, 2021 [82]

Acronyms: DIW—direct ink writing; DLP—digital light processing; SLA—stereolithography; SA—sodium alginate; PEG-DA—poly(ethylene glycol) diacrylate; PAA—poly(acrylic acid); PEG-MA—poly(ethylene glycol methacrylate); PAm—polyacrylamide; TGase—transglutaminase; GOx—glucose oxidase; HRP—horseradish peroxidase; ADH—alcohol dehydrogenase; BFD—benzoylformate decarboxylase; β-Gal—β-galactosidase; ALP—alkaline phosphatase; CAT—catalase; PTE—phosphotriesterase; sfGFP—superfolder green fluorescent protein; 3DJW—3D jet writing; MEW—melt electrowriting; PCL—polycaprolactone.

## Data Availability

Not applicable.
